# Direct laser-writing of ferroelectric single-crystal waveguide architectures in glass for 3D integrated optics

**DOI:** 10.1038/srep10391

**Published:** 2015-05-19

**Authors:** Adam Stone, Himanshu Jain, Volkmar Dierolf, Masaaki Sakakura, Yasuhiko Shimotsuma, Kiyotaka Miura, Kazuyuki Hirao, Jerome Lapointe, Raman Kashyap

**Affiliations:** 1 Department of Materials Science and Engineering, Lehigh University, 5 East Packer Avenue, Bethlehem, PA 18015, USA; 2 Department of Physics, Lehigh University, 16 Memorial Drive East, Bethlehem, PA 18015, USA; 3 Office of Society-Academia Collaboration for Innovation, Kyoto University, Goryo-ohara 1-30, Kyoto 615-8245, Japan; 4 Department of Material Chemistry, Kyoto University, Katsura, Nishikyo-ku, Kyoto 615-8510, Japan; 5 Department of Engineering Physics, Polytechnique Montreal, Montreal, Quebec H3C 3A7, Canada; 6 Department of Electrical Engineering, Polytechnique Montreal, Montreal, Quebec H3C 3A7, Canada

## Abstract

Direct three-dimensional laser writing of amorphous waveguides inside glass has been studied intensely as an attractive route for fabricating photonic integrated circuits. However, achieving essential nonlinear-optic functionality in such devices will also require the ability to create high-quality *single-crystal* waveguides. Femtosecond laser irradiation is capable of crystallizing glass in 3D, but producing optical-quality single-crystal structures suitable for waveguiding poses unique challenges that are unprecedented in the field of crystal growth. In this work, we use a high angular-resolution electron diffraction method to obtain the first conclusive confirmation that uniform single crystals can be grown inside glass by femtosecond laser writing under optimized conditions. We confirm waveguiding capability and present the first quantitative measurement of power transmission through a laser-written crystal-in-glass waveguide, yielding loss of 2.64 dB/cm at 1530 nm. We demonstrate uniformity of the crystal cross-section down the length of the waveguide and quantify its birefringence. Finally, as a proof-of-concept for patterning more complex device geometries, we demonstrate the use of dynamic phase modulation to grow symmetric crystal junctions with single-pass writing.

The ability to fabricate single crystals of desired size and quality has enabled new technologies and often revolutionized solutions of major engineering problems. Examples include silicon crystals that enabled the microelectronics revolution, quartz crystals that formed the basis of precision clocks and resonators, superalloy crystals that dramatically prolonged the life of jet engines, and sapphire crystals that brought complex optics to consumers. Single-crystals now play a key role in telecommunications technology as substrates for nonlinear-optic waveguides. The development of novel approaches to single-crystal fabrication could thus create new paradigms in device design and advance the capabilities of optical communication systems.

Modern telecommunications infrastructure relies on networks of optical and optoelectronic device elements such as waveguides, splitters, modulators, filters, and amplifiers in order to transport and manipulate optical signals^1^. A major trend in optics has been a drive toward integration; that is, replacing systems of large discrete components that provide individual functions with compact and multifunctional photonic integrated circuits (PICs), in much the same way that integration of electronics has driven the impressive advances of modern computer systems^2^,^3^. Such devices can offer several advantages compared to discrete systems including smaller size, lower power consumption, better performance and reliability through simplification of component coupling and packaging processes, and lower cost through batch fabrication^2^,^3^. However, the strict geometric tolerances and unique physical processes involved with manipulating photons introduce a host of new challenges that were not applicable to integration of electronics. Significant progress been made, but the methods currently employed for fabricating PICs are photolithographic processes suitable for planar geometries. 3D PIC fabrication techniques would enable a much higher density of components and much more compact devices^1^, while at the same time creating opportunities for new technologies such as high density 3D optical memory.

The development of ultrafast femtosecond (fs) lasers introduced a promising route for producing 3D integrated devices^1^,^4^,^5^,^6^,^7^,^8^. These lasers are capable of inducing a wide range of local structural modifications when focused inside transparent materials^9^,^10^,^11^,^12^, enabling direct 3D patterning of features inside monolithic samples. Direct writing of amorphous waveguides in glass via fs laser-induced densification has attracted particular interest and yielded promising results^1^,^4^,^5^,^6^,^7^,^8^. Nevertheless, amorphous waveguides fundamentally lack second-order optical nonlinearity due to their isotropically disordered atomic structure, so certain photonic applications that not only transport but also manipulate photonic signals, such as modulators and wavelength converters, require crystalline substrates with second-order nonlinear optical response. With appropriate doping, such crystals are also useful as gain media or laser materials. The ability to pattern nonlinear optical crystals in glass is therefore essential for 3D laser-fabrication of PICs to achieve its full potential.

Patterning of microscopic crystals inside glass by femtosecond (fs) laser irradiation was first reported in 2000 by Miura *et al.* and has since been replicated in several nonlinear crystals and glass systems, with nonlinear-optical activity confirmed by observance of second-harmonic generation (SHG)^9^,^13^,^14^,^15^,^16^,^17^,^18^. These were encouraging first steps, but the significantly more challenging tasks of fabricating single crystals large enough for a functional device and confirming waveguiding capability have remained elusive. Moreover, the morphologies reported in the literature of laser-patterned micro-crystals in glass have been observed mainly by non-destructive optical techniques. Although these results have often revealed a preferential lattice orientation, they have not provided sufficient resolution of the interior microstructure of the crystal to assess single-crystallinity with confidence. As such, and since no examples of waveguiding in fs laser-patterned crystal lines have been reported thus far, the possibility of creating high-quality single crystals capable of waveguiding with this method has remained an open question. Furthermore, when high quality is a concern, extending this method from isolated individual waveguides to more complex connected geometries presents a major challenge. Here we begin addressing these issues, which must be resolved before the technique can achieve practical application.

In this report we demonstrate for the first time the feasibility of single-crystal-in-glass waveguides written by femtosecond laser irradiation with 3D space-selectivity (i.e. arbitrary focal depth). In the first section we utilize electron backscatter diffraction (EBSD) to analyze crystal orientation and morphology at high spatial and angular resolution in the model ferroelectric lanthanum borogermanate (LaBGeO_5_) system, and we verify that high-quality single-crystal lines lacking grain boundaries can be produced by fs laser irradiation under particular conditions. We show waveguiding through such a single crystal and quantify its power transmission in the second section. In the third section, we illustrate the use of dynamic phase modulation to continuously grow a symmetric crystalline splitter via single-pass writing. Dynamic phase modulation greatly expands the possibilities for patterning complex geometries with a single beam^19^, and it can be applied simultaneously with dynamic aberration correction to maintain consistent focal conditions over varying focal depth, enabling fully 3D fabrication^20^.

## Results

### Single-crystallinity

Electron backscatter diffraction provides detailed images of crystal morphology via a color-coded mapping of local crystallographic orientation at each pixel. Individual grains thus appear as regions of consistent coloration. The grain structure can be further revealed by applying a grayscale mask corresponding to diffraction pattern image quality, such that disordered regions like grain boundaries, cracks, scratches, and other surface defects appear black. For the sake of comparison, we include EBSD results for two laser-crystallized lines of ferroelectric LaBGeO_5_, grown by scanning a focused fs laser through a glass of the same composition under different sets of fabrication conditions.

**Figure 1:**
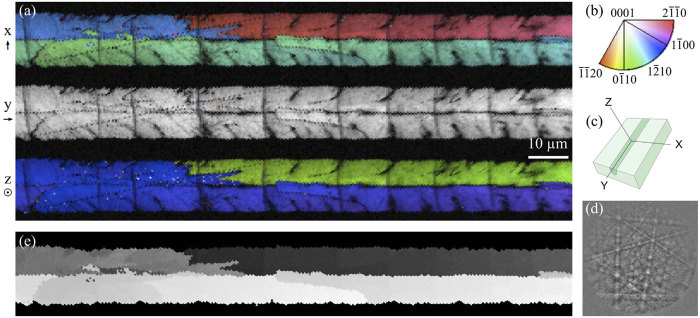
EBSD results for a polycrystal line grown at 25 μm/s scan speed and 500 mW average power, with no aberration correction. Crystal orientation IPF maps overlaid with grayscale image quality masks are given in (**a**) with respect to the three orthogonal axes indicated to the left of each map. The color correspondence of crystal orientation parallel to each reference axis is given in (**b**), and a schematic of the reference coordinate system relative to the sample geometry is given in (**c**). Inversion averaging was disabled in the OIM software in order to highlight the 180° twin (red region in the x-map). An example diffraction pattern is included in (**d**), and (**e**) shows the low-angle grain structure in the x-oriented IPF map, converted to grayscale and contrast-enhanced.

Figure 1 shows EBSD results for a line written under irradiation conditions that were not optimized for single-crystal growth (see caption for details), which clearly exhibits polycrystallinity. Three inverse pole figure (IPF) orientation maps for the same line are considered in (a), each color-coded according to the color map in (b) to display the crystallographic direction oriented along one of three orthogonal reference axes. These axes are defined with respect to the sample geometry according to the schematic in (c): the y direction is parallel to the crystal line, the z direction is normal to the sample surface, and the x direction is orthogonal to both (this is also indicated to the left of each map in (a)).

Note that mapping with respect to different reference directions merely displays different aspects of the same orientation data, and these maps do not represent separate measurements. However, mapping against multiple reference axes uniquely identifies the orientation at each point; any individual map does not distinguish rotations of the lattice about its reference axis. Such a rotation occurs in this case about the y-axis: the entire y-map displays a uniform orientation near [0001] (i.e. the optic axis is approximately parallel to the crystal line), yet the orthogonal x- and z-maps exhibit a distinct grain structure. In particular, the red (x) and green (z) grain represents a twin with an approximately 180° rotation about the [0001] axis with respect to the rest of the line. This is an example of a line that is highly *oriented*, but is not a single crystal.

An example diffraction pattern is given in Fig. 1(d) to illustrate the high pattern quality that was obtained for this phase, enabling precise determination of crystal orientation and resolution of very small misorientation angles. However, such small angle differences are difficult to discern in IPF maps since they appear as subtle changes in hue. Converting an IPF map to grayscale and applying a contrast enhancement can make such low-angle grain structure more pronounced, as shown in (e).

**Figure 2:**
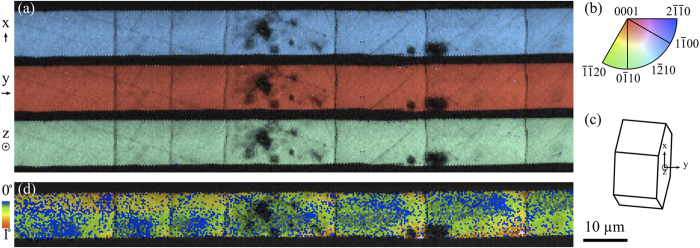
EBSD results for a single-crystal line grown at 42 μm/s scan speed and 300 mW average power, with aberration correction applied. Crystal orientation IPF maps overlaid with grayscale image quality masks are given in (**a**) with respect to the same reference axis defined in Fig. 1. The color correspondence of crystal orientation parallel to each reference axis is given in (**b**), and an illustration of the lattice orientation (represented by a hexagonal cell) is given in (**c**). The angular deviations from the average orientation are mapped in (**d**) to confirm the absence of low-angle grain boundaries.

Figure 2 gives EBSD results for a section of the crystal waveguide discussed in the following section, written under conditions conducive for single-crystal growth (see caption for details). Lattice orientation maps are given in (a) for the same three reference axes, with a color correspondence according to (b). The y-map again shows a consistent orientation of approximately [0001] parallel to the line axis; but in this case, unlike Fig. 1, the x- and z-maps are equally uniform. Altogether this corresponds to the single orientation illustrated in (c) for a hexagonal unit cell. Whereas the grayscale image quality mask of the polycrystal revealed dendrite-like lobes along its edges and internal grain boundaries, both of which should be detrimental to optical transmission, the grayscale features in Fig. 2 are limited to faint scratches, surface debris, and transverse cracks—features introduced during the polishing required to prepare the sample for EBSD, and not representative of the as-made crystal. The coloration is uniform enough that the contrast-enhancement procedure applied in Fig. 1(e) fails to resolve any features in this case, so an alternative approach was used to search for low-angle misorientations (d). Here the color range represents the magnitude of angular misorientations with respect to the average orientation of the line, which demonstrates that the crystal orientation is uniform to within 1°.

### Waveguide characterization

**Figure 3:**
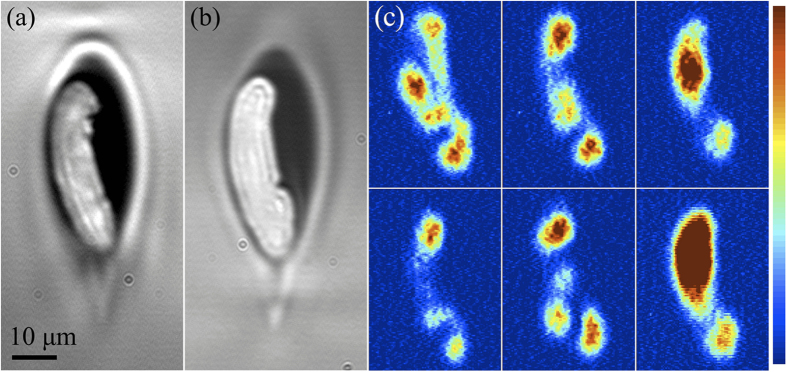
Optical transmission micrographs of input (**a**) and output (**b**) faces of the sectioned crystal-in-glass waveguide, and output power distributions produced by varying the position of the input fiber (**c**).

Figure 3(a) and (b) show cross-sections of the input and output faces (left and right, respectively) of the nonlinear optic LaBGeO_5_ single-crystal waveguide from Fig. 2. The crystal extends through an elliptical channel of visibly laser-modified glass. Near-field intensity profiles of light guided through the waveguide are shown in Fig. 3(c). The various outputs correspond to slightly different positions of the fiber supplying the input 1530 nm wavelength light. The variation indicates that the waveguide can support multiple guided modes. The output in the last frame exhibits substantial transmission of optical power.

In the arrangement which yields the largest power transmission, a 300 mW input yields a 165 mW output over a 0.707 cm length, corresponding to a total loss of 3.67 dB/cm. This value may include contributions from coupling and reflection losses at the interfaces in addition to the loss of the waveguide itself. The reflection contribution can be accounted for by calculating the Fresnel equation for multiple internal reflections, which gives a remaining loss of 2.64 dB/cm (using 1.8 as the transverse refractive index of LaBGeO_5_ at 1530 nm^21^).

Coupling loss is expected if the maximum angle of the fiber’s conical output exceeds the maximum acceptance angle of the waveguide. Both angles depend on their respective numerical apertures (NA) according to


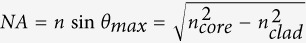


where the index of the medium *n* in this case is 1. The NA of the input fiber is 0.14, which corresponds to an output angle of 8.0° from the beam axis. The core and cladding indices of the LaBGeO_5_ waveguide at 1530 nm are 1.8 and 1.792 respectively^21^, yielding NA of 0.17 and an acceptance angle of 9.8°. Since the acceptance angle exceeds the output angle, there should be minimal interfacial coupling loss, so 2.64 dB/cm represents the best estimate of the transmission loss of the waveguide.

The cross-section of the crystal has an asymmetric shape due to the particular growth dynamics that arise from the interaction between the fs laser-induced temperature gradient and the temperature-dependent growth rate inherent to the crystal. In general, a range of possible shapes may be observed for crystals grown under these conditions^20^. The tendency toward asymmetry could be advantageous for producing polarization-maintaining waveguides^22^, but variation in cross-section shape within any individual waveguide should be minimized in order to sustain a consistent set of guided modes and reduce mode-coupling loss within the waveguide. Accordingly, the crystal growth process must be sufficiently controlled and stabilized such that the shape of the cross-section is approaching constant for the full length of the crystal. We demonstrate this situation in Fig. 3, wherein the input and output faces of the crystal are shown to exhibit the same shape.

**Figure 4:**
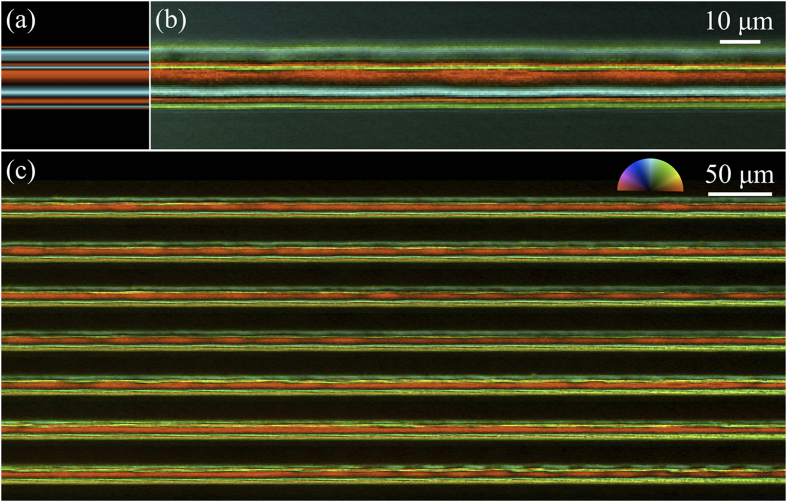
Calculated (**a**) and observed (**b**) LC-PolScope birefringence micrographs of the crystal-in-glass waveguide exhibiting characteristic fringe pattern determined by cross-section shape, crystal birefringence, and lattice orientation. The pattern extends consistently down the length of the waveguide (**c**), indicating a uniform cross-section is retained. The color wheel inset indicates the color correspondence of the angle of either the fast or slow axis of birefringence in the image plane (varying locally depending on crystal thickness; the ambiguity is due to the cyclic nature of the relative phase shift being measured).

Further confirmation that the shape is retained throughout the full length of the crystal was obtained by LC-PolScope imaging, with which the birefringence of the sample was used to nondestructively assess the uniformity of the cross-section geometry and crystal orientation (see the Methods section for details), as well as the absence of cracks prior to polishing for EBSD. Figure 4(a) shows a theoretical LC-PolScope image calculated from the dimensions of the output cross-section in Fig. 3(a), with the birefringence parameter adjusted to maximize agreement between the simulation and the experimentally observed LC-PolScope image in Fig. 4(b). This procedure yields 0.030 as a measure of the birefringence of the crystal. It must be emphasized that the bands of alternating colors do not represent changes in crystal orientation, but result from the varying sample thickness, the cyclic nature of the phase shifts being measured, and the manner in which they are interpreted by the instrument.

The model result in Fig. 4(a) illustrates that a uniform crystal cross-section produces a consistent pattern of bands characteristic to that particular cross-section shape. Likewise, the uniformity of these bands in the collected LC-PolScope micrographs serves as a non-destructive experimental measure of long-range consistency of the crystal cross-section. PolScope micrographs of several sections of the waveguide are shown in Fig. 4(c). A single pattern consistent with the model is represented throughout, indicating that the cross-section shape in Fig. 3 is sustained uniformly along the waveguide. Small fluctuations do occur, suggesting that the crystal varies slightly in size but retains approximately the same shape. Presumably, if these fluctuations were minimized (e.g. by further stabilizing stage motion and beam conditions), waveguide loss could be substantially reduced.

Finally, we note that a birefringence value of approximately 0.04 is reported in the literature for the bulk LaBGeO_5_ crystal^21^, but the LC-PolScope analysis suggests that the value is 0.030 for our crystal-in-glass waveguide. The discrepancy could be associated with residual stresses caused by density and thermal expansion mismatch between the crystal and glass phases.

### Crystal Y-junctions

As the next step in demonstrating the feasibility of laser-patterned 3D-PIC, we turn our attention to the creation of crystalline splitters (Y-junctions). In this, we face a challenge in obtaining symmetric and uniform splitting of the crystal into two diverging paths. The simplest approach of writing first one branch and then the other (two-pass writing) introduces asymmetry between the two branches: the first is grown continuously as an extension of the original line, but the second is initiated discontinuously at its surface, with consequent inhomogeneity at the point of intersection. Simultaneous single-pass writing of both branches is required to achieve symmetric growth conditions and continuity of the crystal across the junction.

**Figure 5:**
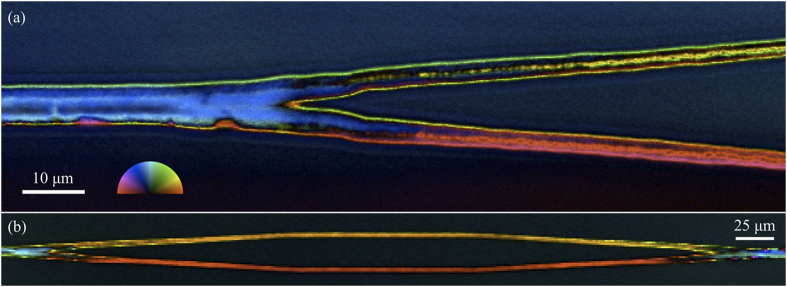
LC-PolScope birefringence micrographs of crystal junctions written inside glass by femtosecond laser with dynamic phase modulation. (**a**) Upon divergence, independent lattice orientations develop in each branch with the optic axis (slow axis) orienting approximately parallel with the growth direction. (**b**) The new orientations are retained across additional changes in growth direction through parallel and converging regions as the branches are merged back to a single line. As in Fig. 4, the color wheel inset indicates the angle of the fast or slow axis of birefringence.

Single-pass writing of complex patterns can be achieved through the use of a digital spatial light modulator^19^,^23^,^24^, which uses an array of phase shifts to create arbitrary beam shapes that can be modified as the focal point is moved through the sample to produce complex dynamic effects. We obtained a gradual splitting of two foci at a 10° divergence angle by rapidly cycling through a series of 112 phase patterns, each of which was calculated to yield a pair of foci at a particular separation distance. The LC-PolScope micrographs of nonlinear optic LaBGeO_5_ crystal junctions in Fig. 5 show the result of applying this procedure during crystal growth.

The orientation of the crystal’s optic axis affects its coloration in LC-PolScope micrographs. In the diverging region shown in Fig. 5(a), the upper branch adopts a yellow coloration indicating that the optic axis has developed an ascending slope, while the lower branch adopts a crimson coloration consistent with the optic axis developing a descending slope. The original blue coloration is consistent with an approximately parallel orientation of the optic axis with the initial path of the line, as seen in EBSD. The crystal lattice orientation thus diverges symmetrically along with the line itself, as the optic axis in each branch attempts to realign itself to become parallel with the new growth direction.

The transition from blue to yellow (upper branch) and crimson (lower branch) at the junction indicates a decrease in crystal thickness. This occurs because the crystal growth is driven by localized heating around the laser focus, and the heat source produced by the individual focus in either branch is weaker than their combined effect prior to divergence. Such a change in cross-section size is undesirable since it will introduce a corresponding change in guided modes and associated mode-coupling losses. However, in principle it should be possible to retain the original thickness in each of the branches by coordinating the dynamic phase modulation with a compensatory power modulation of the laser source.

Once diverged, the two branches may be grown in parallel or manipulated independently through additional phase modulation. Figure 5(b) shows an example which was diverged, grown in parallel, and then converged back to a single line, a configuration used for interferometers. The same trend in optic axis orientation seen in (a) again occurs, with a comparable angular variation (color difference) arising between the ascending and descending slopes. Surprisingly, only the initial change in growth direction at the point of divergence induced a reorientation of the crystal in each branch; the new orientations (colors) were then sustained through the parallel region and even the merging region. In other words, despite multiple changes in growth direction during the course of patterning this structure, only the first change appears to have induced a reorientation of the optic axis toward the new growth direction; presumably by formation of new grains at the intersection region. Each branch of the structure then retained a distinct orientation determined by the initial angle.

## Discussion

The ability to pattern crystalline features and waveguides in glass greatly expands the potential capabilities of compact laser-patterned PICs by enabling incorporation of second-order nonlinear-optical functionality. We have confirmed that high quality single crystals of substantial length can indeed be produced by fs laser irradiation inside glass with 3D selectivity, using a characterization technique with sufficiently high spatial and angular resolution to conclusively rule out low-angle grain boundaries (Fig. 2). At the same time, our results emphasize the necessity of using such a technique to reliably identify single crystals: In the general case represented by Fig. 1, crystals can appear highly oriented along certain crystallographic directions yet retain a distinct grain structure containing rotations and low-angle misorientations with distinct grain boundaries.

Additionally, we have provided the first confirmation and quantitative assessment of waveguiding through fs laser-induced crystals in glass. The loss upper limit of 2.64 dB/cm is a promising starting point that may already be sufficient for nonlinear optical manipulation in PICs that require relatively short transmission lengths (e.g. on the order of mm or cm), and the efficiency can likely be improved with further optimization of fabrication conditions.

The main optimizations used presently include *in situ* annealing, aberration correction, tuning the average power, and manipulating the focal scan rate. *In situ* annealing (by irradiating inside a heated sample stage) helps to suppress cracking, which would otherwise occur frequently due to the stresses associated with thermal expansion and phase change. Aberration correction normalizes beam conditions independent of focal depth and provides more efficient heating, producing a more concentrated heat source and a wider melt zone. This appears to promote more stable and uniform crystal growth by enabling the crystal to grow farther away from the extreme temperatures and explosive pressures induced by the high intensity fs laser pulses at the focus. Increasing the average power of the laser has a similar effect, but also caused a stronger tendency to crack at higher power that could not be prevented by *in situ* annealing. The power was thus adjusted to strike a balance between these effects.

The speed of scanning the laser focus through the glass appears to be the key parameter for establishing single-crystal waveguides. Crystals could be sustainably grown over a range of scan speeds, but generally developed polycrystalline morphologies. At speeds above this viable range, the growth front would become unable to keep pace with the heat source, eventually falling behind and ceasing to grow any further. Growth could still proceed for some time at scan speeds slightly above this threshold, but only with a gradual tapering of the cross-section that could not be sustained indefinitely. Interestingly, this situation appears to promote single-crystallinity at the tip of the taper.

By way of explanation, we propose that scanning the focus at slightly supercritical speeds introduces a sort of ‘selection pressure,’ driving a competitive process wherein grains of the polycrystal are progressively filtered out according to their growth rate in the direction of scanning. Growth rate depends on several factors including the local temperature and composition at the growth front and the crystallographic orientation of the grain. The grains whose configurations produce the slowest growth in the scan direction would be the first to fall behind while faster grains would persist longer. By the time the line tapers to a stop, often only a single grain remains: one whose configuration yields the highest growth rate in the direction of scanning.

Once such a grain was produced, it could then be extended indefinitely by reducing the scan speed to just below its maximum sustainable growth rate. We took advantage of this by writing waveguides with a two-pass method: a first pass at high speed to create a tapered crystal, and a second pass at slightly reduced speed to extend it into a single-crystal line of arbitrary length. Our system was only equipped for writing at fixed speeds, but in general it should be possible to modulate scan speed in order to obtain the same effect in a single pass. We also note that there is a relationship between the average laser power and the optimal writing speed^8^, and the maximum speed of crystal growth was reduced at lower power.

Finally, we have also shown that symmetric crystalline junctions for splitters or modulators can be patterned in a single pass by phase modulation of a single beam. These crystal junctions demonstrate the generalization of the technique to more complex structures beyond straight lines; by coordinating sample stage motion with phase modulation (and potentially power modulation), device elements requiring complex continuous geometries involving bends, curves, and junctions can be achieved. Furthermore, the simultaneous application of aberration correction ensures that consistent crystallization conditions can be produced at arbitrary focal depths^20^, a key consideration for 3D fabrication.

While the present results were size-limited by the capabilities of our equipment, it should be noted that all presented approaches are scalable, and no barrier could be identified for the scale-up to longer crystals in a commercial environment. The critical element that must be established is the ability to reliably control the shape and size of the cross-section and retain uniformity over long distances. Application of more complex phase modulation to reshape the temperature distribution will likely be instrumental in this regard, as well as maximizing stability (i.e. smooth stage motion, vibration prevention, and very stable beam conditions).

## Methods

### Laser System

Crystalline features were fabricated using a regeneratively amplified Ti:sapphire femtosecond laser oscillator with 800 nm wavelength, 250 kHz repetition rate, and 130 fs pulse width. A 50× magnification, 4 mm focal length, 0.55 numerical aperture objective lens (Nikon CFI LU Plan EPI ELWD) was used to obtain a sufficiently long working distance (10.1 mm) to enable irradiation within a heated sample stage. Seed crystals were initiated at room temperature, but samples were held at 500 °C during waveguide and junction writing to suppress cracking during crystal growth. Waveguides were initialized from seed crystals by scanning the focus at 46 μm/s, slightly higher than the sustainable growth rate, in order to obtain an optimally oriented single crystal. The speed was then reduced to 42 μm/s to achieve stable, sustainable growth.

A digital liquid-crystal-on-silicon spatial light modulator (LCOS-SLM) was used to apply variable beamshaping, including aberration correction for all samples and focal divergence for the junctions. The modulator consisted of an array of 600 × 792 pixels, each of which could apply a variable phase modulation between 0 and 2π. The phase shifts to be applied were supplied as grayscale bitmaps to a homemade control software, which was designed to accept a series of sequential patterns and cycle through them at a fixed rate. The procedure for calculating the phase patterns for writing single-pass junctions is described in the following section. These were summed with aberration correction patterns to achieve aberration correction during junction fabrication.

For more details regarding optical setup, preparation of LaBGeO_5_ glass samples, initiation of seed crystals, and implementation of aberration correction, we refer the reader to our previous paper^20^.

### Junction fabrication

**Figure 6:**
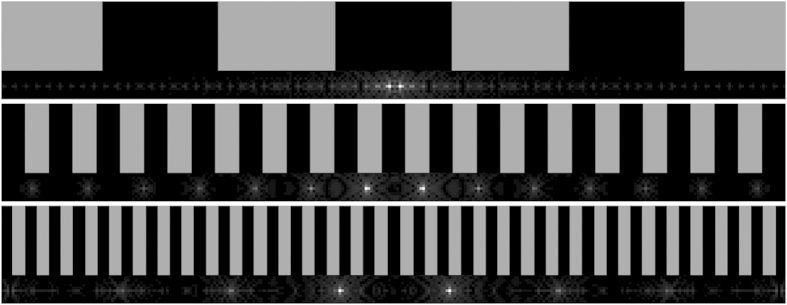
Sections of SLM hologram gratings (above) and their calculated focal intensity distributions (below), illustrating the effect of grating period on focal separation distance (only the two central foci were used in crystallization of the junctions).

Crystalline junctions were written in a single pass by using phase modulation to gradually diverge a pair of foci while scanning the sample laterally with the motor stage after crystal growth had been established^19^. In general, a Gerchberg-Saxton algorithm^24^ can be used to calculate an SLM phase pattern that will produce a particular distribution of optical intensity within the focal plane (e.g. multiple foci), and the obtained pattern may be summed (mod 2π) with an aberration-correction pattern to obtain a combined effect. For the special case of a pair of foci symmetric about the center, a phase pattern consisting of a simple diffraction grating of alternating π-shifted bands (Fig. 6) can be used. The relationship between the period of the grating, *p*, and the real-space distance between the foci, *d*_*foci*_ is given by


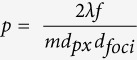


where *p* refers to the number of pixels on the SLM spanned by a pair of adjacent bands, *d*_*px*_ is the SLM pixel size (20 μm), *m* is the magnification of the telescope optics inside the SLM module in the direction of beam propagation (0.5), λ is the laser wavelength, and *f* is the focal length of the objective lens (4 mm). For writing crystal junctions, a gradual divergence of two foci was implemented by cycling through 112 individual phase gratings, symmetric about the center and with periods ranging between 32 and 286 SLM pixels in order to give a linear variation in distance between foci ranging from 2.24 to 20 μm.

As *d*_*foci*_ increases, the grating period decreases, such that eventually the period changes by less than one pixel per step. For example, the last four gratings of the total 112 had periods between 32 and 32.8, corresponding to distances between foci ranging from 19.52 to 20 μm. In order to approximate such fractional changes in grating period, since the SLM is limited to discrete pixels, the width of individual bands was varied such that the average period approached the fractional value.

The crystal was found to follow more reliably into both branches when relatively low scan speeds were used, so the sample was scanned at 10 μm/s. The phase gratings were cycled at a rate of 8 frames per second. Two closely-spaced foci were used as a starting point rather than a single unmodified focus, because this was found to facilitate continuous growth across the junction. The starting distance of 2.24 μm was small enough to produce a single line, but this split into two lines as the foci diverged, each at an angle of approximately 5° from the initial line axis. The two branches were then grown in parallel for some time at 20 μm spacing before the sequence was reversed in order to merge the two branches back into a single line.

### Electron Backscatter Diffraction

Electron backscatter diffraction (EBSD) is a scanning electron microscopy (SEM) technique which provides a detailed probe of local crystallinity, including both phase identification and lattice orientation with high angular resolution, and relatively high spatial resolution (on the order of backscattered electron imaging). EBSD data were acquired in a field-emission SEM in variable pressure mode (enabling analysis of uncoated non-conducting samples) and processed in the Orientation Imaging Microscopy (OIM) Data Analysis software.

EBSD results are generally displayed as inverse pole figure (IPF) orientation maps, in which crystal orientations are described relative to some frame of reference defined by three orthogonal axes related to the sample geometry (e.g. one normal to the surface, one parallel to the surface pointing in the tilt direction, and one orthogonal to both). A color mapping is used to indicate the crystal lattice orientation which lies parallel to any reference direction of interest defined within this frame. Individual grains can be distinguished as regions of consistent color. Other microstructural features were emphasized by applying a grayscale image quality mask. Here “image quality” refers to the sharpness and contrast of the obtained diffraction pattern, so an image quality mask darkens pixels where no clear diffraction pattern was obtained such as amorphous regions, grain boundaries, surface debris, and polishing scratches. Also, because the crystalline phase considered here can exhibit an ambiguity in distinguishing a unique result between certain twin orientations, a pseudosymmetry correction was applied to IPF maps that exhibited significant pseudosymmetry artifacts (i.e. scattered noise-like pixels of particular orientations that lack any appearance of grain structure or grain boundary features in image quality masks).

In addition to IPF orientation maps, we used two approaches to emphasize low-angle grain boundaries. The first was a simple image processing, in which an IPF map was converted to grayscale and a level adjustment was applied to maximize contrast. This had the effect of emphasizing subtle changes in hue corresponding to low-angle misorientations, which were difficult to discern in the original IPF map. The second method was applied in cases where the first failed to reveal distinguishable features, as was the case with single crystals. In this method, a spectral color-mapping was used to indicate the deviation from the average orientation of the grain at each pixel. The range of misorientations could be tuned to encompass the full range of misorientations occurring in the crystal, such that the angular resolution was maximized.

### LC-PolScope Imaging

An LC-PolScope is a specialized optical microscope equipped for detailed analysis and imaging of sample birefringence. The alternating colored bands in the LC-PolScope micrographs in Figs. 4,5 are related to the vertical thickness of the crystal, the orientation of the crystal lattice, and the magnitude of birefringence^23^. A simple model for approximating the pattern of bands associated with a given cross-section geometry and birefringence value was used in the analysis of LC-PolScope results. The model assumes vertical light transmission through the crystal (i.e. a collimated beam with negligible refraction effects at the glass-crystal interface), which is a reasonable approximation since the refractive index difference between the crystal and glass is small^21^.

In this procedure, an image of the cross-section (as in Fig. 3) was converted to a binary matrix with pixels outside the crystal set to 0 and pixels inside the crystal set to 1. The sum of each column was then taken, yielding a 1D array of values proportional to the crystal’s vertical thickness (d), with a proportionality constant determined by the scale bar of the image. This was multiplied by an adjustable birefringence parameter (Δn) to obtain a 1D plot of retardance (Γ) according to Γ = dΔn. A modulo π operation was applied to mimic the LC-PolScope imaging algorithm, which considered only values between 0 and π due to the cyclic nature of retardance (producing the appearance of bands). The 1D plot was then projected into a 2D map to simulate the retardance pattern that would be obtained by the LC-PolScope when imaging a crystal line with perfectly uniform cross-section. Finally, a color-map was applied to reproduce the oscillation between orthogonal slow-axis orientations that occurs in the LC-PolScope images at retardance multiples of π due to the inherent ambiguity in distinguishing fast and slow axes (since the LC-PolScope only detects a relative phase shift). The particular colors correspond to a parallel alignment of the optic axis with the line, which was confirmed by EBSD. The birefringence parameter was adjusted to produce the best agreement between calculation and experiment.

### Waveguide characterization

The waveguide-containing sample was sectioned at both ends with a diamond wafering blade, ground with SiC paper, and polished with CeO_2_ slurry. A 1530 nm wavelength continuous-wave laser at an output power of 300 mW was delivered to one face of the crystal waveguide through a standard Corning SMF28 single mode fiber with 0.14 NA and 10.5 micron mode field diameter, which was aligned to the waveguide with the aid of an optical microscope and analog stage controls. No refractive index-matching oil, anti-reflection coating, or lens system to match the waveguide numerical aperture was used. The near-field waveguide output was imaged through a 20x objective lens and CCD camera. For power measurement, the CCD was replaced with a power meter, and an aperture was used to cut off scattered light and isolate the guided output. The input position was optimized to maximize the measured power.

## Author Contributions

Crystallization experiments and LC-PolScope characterization were performed by A.S. with the assistance of M.S. and Y.S. and supervision of K.M. and K.H. Waveguide characterization was performed by J.L. and A.S. with supervision of R.K. Manuscript was prepared by A.S. with input from all authors. H.J. and V.D. guided the overall project.

## Additional Information

**How to cite this article**: Stone, A. *et al*. Direct laser-writing of ferroelectric single-crystal waveguide architectures in glass for 3D integrated optics. *Sci. Rep.* **5**, 10391; doi: 10.1038/srep10391 (2015).
